# Localised 0.2% chlorhexidine irrigation delivery system versus oral antibiotics in reducing postoperative complications in the surgical extraction of impacted mandibular third molar (IMTM). - a randomised controlled trial

**DOI:** 10.4317/medoral.26676

**Published:** 2024-08-18

**Authors:** Ankita Chugh, Gigi P.G, Pravin Kumar, Amanjot Kaur

**Affiliations:** 1 Oral and maxillofacial Surgery, Department of Dentistry, All India Institute of Medical Sciences, Jodhpur, Rajasthan, India; 2 Department of Dentistry, All India Institute of Medical Sciences, Jodhpur, Rajasthan, India; 3 Oral and maxillofacial Surgery, All India Institute of Medical Sciences, Vijaypur, Jammu, India

## Abstract

**Background:**

The purpose of the study was to compare the efficacy of the use of 0.2% chlorhexidine irrigation and the oral antibiotics for the prevention of postoperative complication like pain, trismus, swelling and infection after the surgical extraction of IMTM.

**Material and Methods:**

A randomised, double blinded clinical trial was planned with two equal groups. Patients were randomly divided into two groups using computer-generated codes with an allocation ratio of 1:1. Group I (Control): Standard preoperative and postoperative systemic oral antibiotics and Group II (Study): No systemic antibiotics and Chlorhexidine irrigation local delivery. The primary outcomes evaluated were postoperative pain, mouth opening, swelling and infection. The secondary outcome variables were the number of analgesics and antibiotics taken by the patient in the postoperative period, the satisfaction of the patient and adverse events, were followed up regulary for 7 days postoperatively.

**Results:**

A total of 84 patients, divided into two equal groups participated in the study. In intergroup comparison of swelling, the difference was non-significant on postoperative day (POD) 1 and 7, except for POD 3, where it showed significantly lower results in the antibiotic group (*p* = 0.012). However, there was no significant difference in pain found between both groups at any of the postoperative time points, and the study group had a lesser need for rescue analgesics than the control group. A statistically significant difference in incidence of dry socket was observed (*p* = 0.03) and gastrointestinal adverse symptoms, but it showed insignificant results for wound dehiscence and pus discharge. Also, patient satisfaction was higher in the study group.

**Conclusions:**

both antibiotics and localised delivery demonstrated comparable results in terms of swelling, pain and trismus. However, with lesser adverse events, the localised chlorhexidine delivery with curved tips outperformed the antibiotic group.

** Key words:**Surgical extraction. chlorhexidine. antibiotics, complications, impacted third molar.

## Introduction

Surgical extraction of the impacted mandibular third molar (IMTM) can cause minor and major complications, such as pain, swelling, trismus and infection, which can adversely affect patient quality of life (1). These complications are usually attributed to inflammation secondary to the procedure itself, tissue injury or postoperative infection. There have been several attempts in the literature to negate these complications and achieve successful postoperative recovery.

Systemic medications such as antibiotics, glucocorticoids and anti-inflammatory agents; local or topical therapy, including chlorhexidine rinses and gels; and cryotherapy and minocycline are frequently used (2-9). However, these therapies have inherent limitations and are associated with systemic or local adverse reactions. In addition to obvious antimicrobial resistance, antibiotics can cause various adverse drug reactions, such as skin reactions, gastrointestinal alterations, liver problems and hematological complications (10). Thus, researchers must look for other ways of preventing bacteraemia. Previous studies have shown that antibacterial mouth rinse products, such as chlorhexidine, iodophors and phenolics, can effectively decrease the overall quantity of oral bacteria and reduce their negative effects (such as periodontitis), as well as the incidence of bacteraemia induced by various dental procedures (11,12).

Studies have shown that using 0.12% chlorhexidine perioperatively could significantly decrease the incidence of alveolar osteitis (AO) after mandibular third molar surgical extraction(s) (13-15) and some reports have encouraged the use of CHLORHEXIDINE as a rinse for extraction sockets.

However, its action is rapid but not prolonged, and localized action focused on the surgical site area is not warranted. Additionally, its constant use as an oral rinse can generate adverse effects, such as external dental staining, dysgeusia, oral mucosa lesions, and dental calculus formation (16). Localized irrigation of chlorhexidine with specialized curved tips may be more beneficial because it directs action on the surgical site specifically. The use of chlorhexidine, in any formulation (rinse or gel), at any concentration (0.12% or 0.20%), or regimen (before, during and/or after surgery), is efficacious and effective at preventing AO in patients who have undergone third molar extraction (17). However, its role in minimizing other postoperative sequelae is not specifically evident in the literature.

Thus, a randomized controlled trial (RCT) was planned to compare the efficacy of 0.2% chlorhexidine irrigation to that of oral antibiotics in reducing postoperative complications after surgical removal of third molars.

## Material and Methods

- Trial design and ethical approval

A single-center, double-blinded, prospective randomized controlled trial was conducted after ethical approval was obtained from the Institutional Ethical Committee (AIIMS/IEC/2021/3741). This study was performed according to the Declaration of Helsinki regarding medical research, 2016, and was carried out strictly according to CONSORT guidelines (18).

- Settings and Consents

All patients with IMTM visiting the Department of Dentistry of our Institute were analyzed from August 2022 to November 2022, and patients were enrolled in the study after applying the eligibility criteria and providing written informed consent.

- Eligibility criteria

ASA I and II patients aged 18-50 years with IMTM (requiring and willing for surgical extraction) with similar difficulty indices as assessed by the Pell and Gregory classification (19)were included in the study (difficulty level minimal= 3-4, moderate = 5-7 ,very difficult= 7-10). Patients with uncontrolled systemic disease or a history of allergy to local anesthesia or chlorhexidine were excluded from the study.

- Sample size analysis and procedures

The sample size were calculated based on previously published study done by Mariscal-Cazalla *et al*. Assuming (20) a standard deviation of 2.3 in study group and 2.8 in control group with effect size of 0.74 and clinical meaningful mean difference of 1.9 in mean VAS scores at 72 hours after the procedure in two treatment groups, with 90 percent power and alpha error of 5%, the, the sample size was estimated to be 38 per treatment group. Assuming a dropout rate of 10%, 42 patients were recruited per treatment group. For a total of two groups, 84 patients were recruited.

- Randomization and blinding

Patients were randomly divided into two groups using computer-generated codes by an individual “X” with an allocation ratio of 1:1. The codes were sealed in sequentially numbered opaque envelopes to ensure allocation concealment.

Group I (Control Group): Standard preoperative and postoperative systemic oral antibiotics

**Figure 1:**
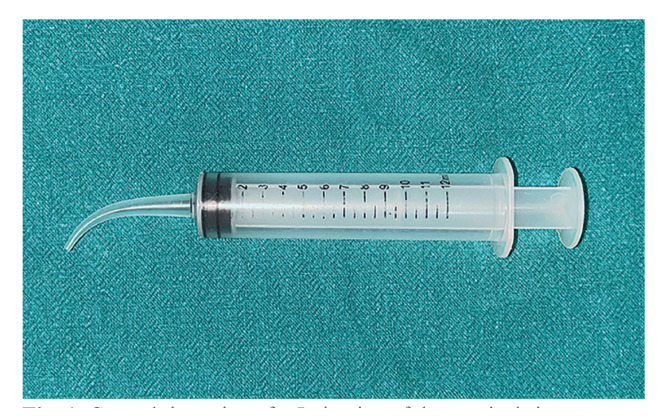
Curved tip syringe for Irrigation of the surgical site.

Group II (study group): Patients were not treated with systemic antibiotics. Chlorhexidine irrigation via local delivery via specialized curved tips (Fig. 1) twice daily during the postoperative period

The assessor/investigator and the surgeon were blinded throughout the study.

- Intervention

Group I patients were given a single dose of a combination of oral antibiotics (Tablet Amoxicillin 500 mg and clavulanic acid 125 mg) 1 hour prior to surgical extraction.

No preoperative antibiotics were given to the patients in the study group. All procedures were performed by a single surgeon. A standardized surgical procedure was adopted for all patients and was performed under sterile conditions. Local anesthesia (2% Lignocaine 1:80000 with adrenaline) was used for inferior alveolar and buccal nerve blocks. A conventional Ward incision was used to establish the mucoperiosteal flap, and surgical exposure/delivery of the tooth by bone removal was performed using a surgical drill under continuous saline irrigation. Socket toileting was performed prior to closure with interrupted sutures with 3-0 vicryl. The postextraction instructions were clearly explained to all patients. Systemic oral antibiotics (Tablet amoxicillin 500 mg and clavulanic acid 125 mg) were administered thrice daily for three days to the control group. No systemic antibiotics were given to the patients in the study groups during the postoperative period until an infection occurred, at which point the dosage was recorded. The study group was administered chlorhexidine via local delivery via specialized curved tips twice daily after meals. Both groups received postoperative analgesics (ibuprofen 400-mg Tablet, paracetamol 333-mg Tablet) for 3 days, 3 times a day. Any patient in the control or study group who required additional analgesic dosages from postoperative day (POD) 4 onward was permitted to do so, and the dosages were recorded.

- Outcome measured

**Figure 2:**
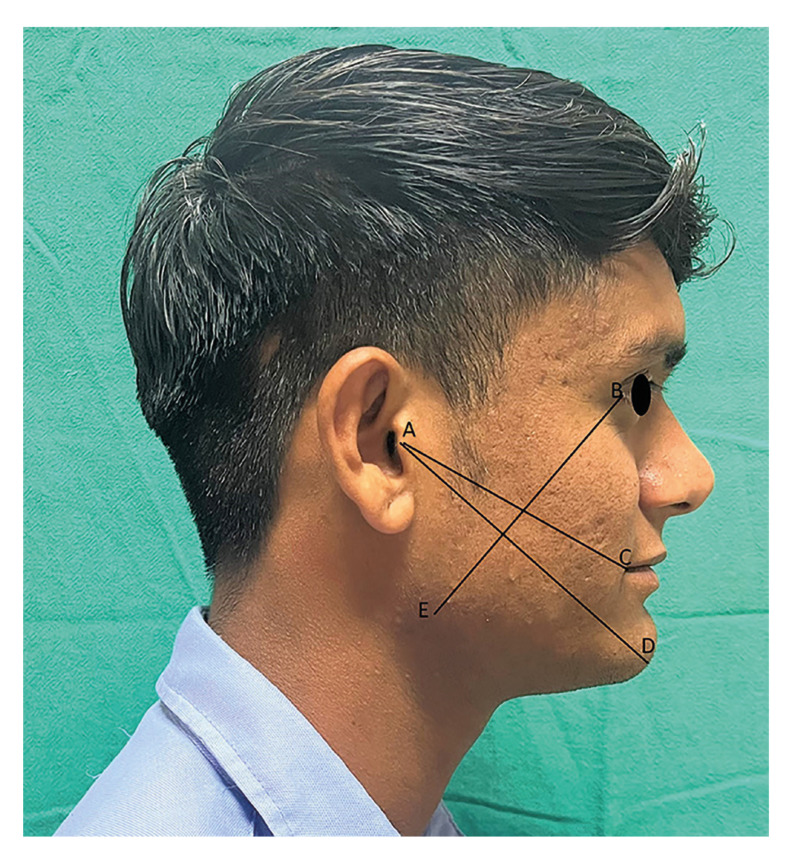
Measurement of facial swelling.

All demographic details were recorded for all patients. The primary outcomes were postoperative pain, mouth opening, swelling and infection. The secondary outcome variables were the number of analgesics and antibiotics taken by the patient in the postoperative period, the satisfaction of the patient and adverse events. Postoperative pain (PODs 1, 3, 5, and 7) was assessed using an 11-point NRS-modified visual analog scale. Interincisal mouth opening/trismus (preoperative, POD 1, 3, 7) was assessed by the doctor as the maximum interincisal opening measured with Vernier calipers in millimeters (mm). Facial swelling (preoperatively, PODs 1, 3, and 7) was assessed in millimeters with flexible measuring tape using the 3 reference planes. AC, from the most posterior point on the tragus to the lateral point on the corner of the mouth AD; from the most posterior point on the tragus to the soft tissue pogonion BE; and from the lateral canthus of the eye to the most inferior point on the mandibular angle (Fig. 2). The total swelling in the AC+AD+BE group was measured as facial swelling at the indicated points.

The resultant facial swelling was calculated as the difference between postoperative and preoperative swelling: postoperative swelling (AC + AD + BE) - preoperative swelling (AC + AD + BE) at the desired time points. The number of analgesics taken from POD 4 to 7 was recorded. The number of antibiotics, if needed, taken from POD 1 to 7 was recorded in the case of the study group. In the control group, the need for any additional antibiotics or antibiotic upgrades was noted. Any incidence of dehiscence, dry socket or pus discharge, or allergy was noted from POD 1 to day 7. The duration of surgery was calculated as the time taken from the incision to the last suture placement. A 5-point Likert scale was used to score patient satisfaction at the end of the 7th day.

- Statistical analysis

The data are expressed as the mean ± standard deviation (SD)/error. Baseline demographic data were analyzed using the chi-square test. Independent Student’s t tests were used for comparisons of preoperative mouth opening, swelling and duration of surgery between the 2 groups. One-way ANOVA was used to compare primary outcome variables. Fisher’s exact test was used to compare complications, and regression analysis was used for patient satisfaction analysis. Analysis was performed using SPSS version 23 (IBM Corp. Ltd., Newark, USA). A *P* value less than.05 was considered to indicate statistical significance.

## Results

**Figure 3:**
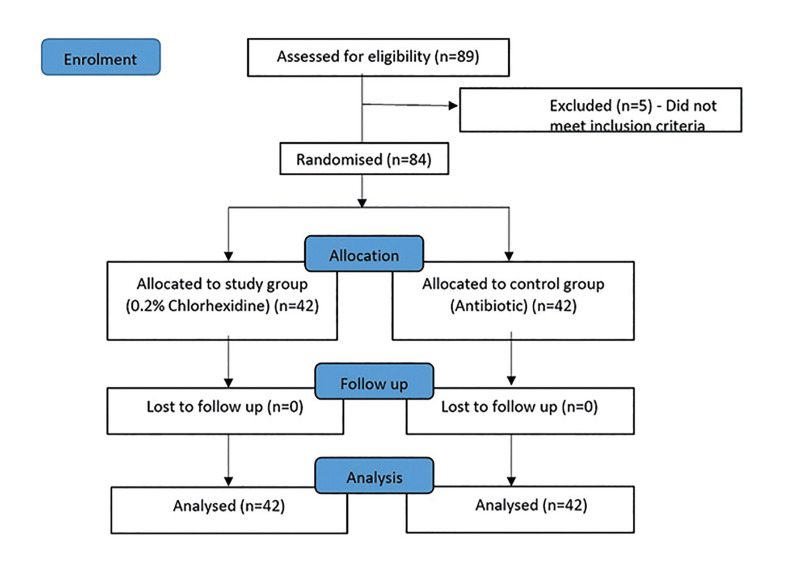
Study flow chart.

The flowchart of the study is shown in Fig. 3. The baseline demographic data, such as age, sex, oral habits, smoking status, use of smokeless tobacco, tooth extraction site and difficulty level, were not significantly distributed in either group, as shown in Table 1.

With regard to postoperative pain during the 7-day follow-up period, the data showed that there was a progressive reduction in VAS score in both groups. There was less pain on POD 1 in the study group. However, there was no significant difference found between the groups at any of the postoperative time points (PODs 1, 3, 5, and 7) (*P*=0.597, 0.400, 0.086, and 0.365, respectively). Although there were no statistically significant differences between the two groups, the study group had a greater need for rescue analgesics than the control group did (Table 2).

Postoperative swelling was significantly greater in both groups than preoperatively. However, in the intergroup comparison, the difference between the two groups was not significant on POD1 or POD 7. On POD 3, swelling was significantly lower in the antibiotics group (*P*=0.012), and on POD 7, residual swelling was significantly lower in the study group than in the control group. Due to pus discharge from the surgical site, 2 patients in the control group still required additional antibiotics after 3 days of antibiotic treatment. Only 2 of the 42 participants in the study group had pus discharge and were given antibiotics.

Her mouth opening, which was recorded on POD1, improved by day seven in both the study and control groups, but the difference was not statistically significant at all time points.

In comparison with the use of antibiotics, chlorhexidine therapy was consistently found to reduce the development of dry sockets after extraction. There were 5 dry socket fractures in the control group versus 1 in the study group (*P*=0.037). There were no significant differences in wound dehiscence (*P*=0.210) or pus discharge (*P*=0.952) between the groups.

Nine patients in the control group experienced a few minor side effects, including nausea (n = 4), gastritis (3), and abdominal discomfort (2), which were promptly treated. The intervention groups reported no significant side effects.

Patient satisfaction was measured using a 5-point Likert scale. In contrast to those in the control group (*n*=2), more patients in the study group (*n*=4) strongly agreed with the treatment modality provided to the study group because of the perception of having better treatment tolerance.

## Discussion

The role of antibiotics in the surgical extraction of mandibular third molars is controversial (21-23) as many SRs have reported mixed results. However, the widespread injudicious use of antibiotics leads to antimicrobial resistance (AMR), which continues to pose a significant loss to public health and the economy. There have been efforts to combat this AMR by searching for alternative methods, such as localized antibiotic delivery systems, chlorhexidine irrigation/rinse/gel, cryotherapy and ozone therapy (24,25) which have been used after surgical extraction of IMTM. This study was a randomized, double-blind, parallel group comparing antibiotics versus chlorhexidine for reducing complications after surgical extraction of IMTM, in which both antibiotics and chlorhexidine had equally efficacious results with fewer adverse events.

Chlorhexidine is a commonly used antimicrobial agent in various forms because of its bactericidal and bacteriostatic effects (26). Chlorhexidine, in any form or formulation, regimen or concentration is efficacious and effective at reducing postoperative complications; however, localized delivery with gel was found to be better than that with rinsing, but poor retention in the oral cavity may lead to a suboptimal therapeutic effect (27). This has led us to use curved tips for localized delivery of chlorhexidine, which, through irrigation of the wound, prevents food lodging, leading to early recovery of erythema and pain from third molar surgery in addition to its pharmacological action due to greater contact at the surgical site.

Swelling or edema is usually a secondary response to tissue manipulation and trauma during surgery. The onset is usually gradual and maximum swelling is usually at 48 hours (28), which tends to resolve from 4th day and complete resolution by 7th day (29).

The difference in the mean preoperative and postoperative facial measurements was not significant except on POD3 (*P*=0.012). On POD1 and POD 7, the intergroup comparison was nonsignificant despite the lack of antibiotic use in the study group. These results are similar to those of previous studies by Cho *et al*., 2017(24), which showed a significant decrease in facial swelling at seven days in the chlorhexidine group but not at 48 hours. Zietler *et al*. also reported that the use of antibiotics slightly decreased trismus, pain and swelling; however, these effects were not significant at follow-up visits (30).

Pain scores were greater in the chlorhexidine group at most time points but were not significantly different between the two groups, although no systemic antibiotics were used to prevent infection or pain exacerbation. On POD 7, the VAS score was lower in the chlorhexidine group. The localized delivery of chlorhexidine leads to the removal of biofilm on the surgical site, which leads to early recovery at 7 days postsurgery. Similar results were found for mouth opening in both groups, with significantly less mouth opening in the study group.

The gastrointestinal effects in the control group (9 patients) were due to the disturbance of the gut flora by antibiotics. Gastrointestinal effects (nausea, vomiting, diarrhea, abdominal pain, loss of appetite) and dermatologic events (rash, hives) are the most common adverse effects of systemic antibiotic use, but many antimicrobial agents have other severe and serious adverse effects (anapospaxis, drug-induced mixed hepatitis, Clostridium difficile infection, etc.) in addition to increased bacterial resistance (31).

The chlorhexidine group showed fewer episodes of dry socket surgery than did the antibiotic group, which has already been proven in the previous literature (32). An important adverse event observed in our study was a high chance of dehiscence in the chlorhexidine group, which was hypothesized to be a result of aggressive insertion of the curved tip at the surgical site by the patient. Thus, we are strictly emphasizing focused individualized training with curved tips for both patients and caregivers. The ability of irrigation to remove debris and deliver chlorhexidine to the surgical site may account for our finding of a lower incidence of dry socket and non-significant pus discharge in the chlorhexidine group compared to the antibiotic group. This minimises the food and debris that stagnates at the operative site and explains the lower incidence of inflammatory complications in the chlorhexidine group.

Patients reported convenience of use and lack of significant problems with the use of curved tips for irrigation. The surgeons could clean the surgical site properly, which made them comforTable. The curved tip irrigation syringes are reusable and very cost effective. Due to the avoidance of antibiotics and their side effects, related cost benefits, the lack of warning of excessive pain, swelling and trismus and better overall patient satisfaction in the chlorhexidine group seem to be similar to those in the antibiotic group. However, the lower overall satisfaction could be attributed to antibiotic-related adverse effects in the control group.

Our study has several strengths, as we strictly adhered to the study protocol with near-perfect randomization of the demographic parameters of both groups, curbing selection bias. The study was double blinded, reducing detection bias. There was no attrition bias in the study, and all participants completed the study. There were no adverse effects in the study group.

Limitations of the study: A subgroup analysis based on difficulty level and duration of surgery could have been performed, and additional extensive and advanced studies at the microbiological and molecular levels to extract additional information could be performed.

## Conclusions

Localized chlorhexidine delivery via curved tips had superior effects on adverse events than delivery via antibiotics. Avoidance of the transient adverse effects of antibiotics by minimizing the chances of antimicrobial resistance and reducing the cost burden with similar clinical results are promising benefits that favor the use of chlorhexidine with targeted delivery at surgical sites using specialized tips in place of antibiotics. Despite the limitations of the study, the localized use of chlorhexidine in curved tips can be recommended compared to the use of oral antibiotics in the surgical extraction of IMTMs for early recovery.

## Figures and Tables

**Table 1:** Baseline demographic data of the participants.

VARIABLES		GROUP I (CONTROL -ANTIBIOTICS) (N=42)		GROUP II (STUDY-CHLORHEXIDINE) (N=42)		*P VALUE*
		FREQUENCY	%	FREQUENCY	%	
AGE	LESS THAN 20	3	7.1	1	2.4	0.895
	20-25 YEAR	17	40.5	15	35.7	
	26-30 YEAR	9	21.4	10	23.8	
	31-35 YEAR	5	11.9	7	16.7	
	36-40 YEAR	3	7.1	4	9.5	
	MORE THAN 40	5	11.9	5	11.9	
SEX	MALE	27	64.3	22	52.4	0.268
	FEMALE	15	35.7	20	47.6	
HABIT	NO HABIT	38	90.5	35	83.3	0.494
	SMOKING	2	4.8	2	4.8	
	TOBACCO	2	4.8	5	11.9	
FREQUENCY OF HABIT	NO HABIT	38	90.5	35	83.3	0.537
	1TIMES	1	2.4	0	0.0	
	2-3 TIMES	1	2.4	2	4.8	
	3-4 TIMES	2	4.8	4	9.5	
	4-5 TIMES	0	00	1	2.4	
TOOTH EXTRACTED	RIGHT	13	31.0	18	42.9	0.538
	LEFT	29	69.0	24	57.1	
DIFFICULTY LEVEL	MINIMALLY	22	52.4	18	42.9	0.651
	MODERATE	15	35.7	17	40.5	
	VERY DIFFICULT	5	11.9	7	16.7	
MOUTH OPENING		MEAN	SD	MEAN	SD	0.328
		38.81	4.06	39.76	4.77	

**Table 2:** Comparison of postoperative parameters between study and control groups.

		GROUP I (CONTROL -ANTIBIOTICS) (N=42)	GROUP II (STUDY-CHLORHEXIDINE) (N=42)	TEST	*P VALUE*
Mean pain score (Mean±SD)	POD 1	7.05± 1.01	6.93 ± 1.04	A	0.597
	POD 3	4.21± 1.02	4.40 ± 1.03	A	0.400
	POD 5	1.64± 0.72	1.93± 0.77	A	0.086
	POD 7	0.62± 0.76	0.48± 0.67	A	0.365
Mean mouth opening (mm)	POD 1	28.90± 3.65	28.81 ± 4.67	A	0.720
	POD 3	31.81 ± 3.14	31.27 ± 4.61	A	0.887
	POD 7	37.57 ± 3.98	36.40 ± 4.71	A	0.413
Mean difference in facial swelling (mm)	POD 1	1.5±1.01	1.74±1.18	B	0.315
	POD 3	0.66±0.78	1.31±1.41	B	0.012
	POD 7	0.09±0.69	0.03±0.91	B	0.751
Total number of analgesics (m Mean±SD)(POD4- POD7)		0.90±1.77	0.71±1.75	B	0.632
Total number of antibiotics(Mean±SD) (POD4-POD7)		0.29±1.29	0.43±1.94	B	0.692
Number of complications	Total	09	09	-	-
	Dehiscence	2	6	C	0.210
	Dry socket	5	1	C	0.037
	Pus	2	2	C	0.952
Adverse events- gastrointestinal symptoms		9	0	-	-
Patient satisfaction scale	Total	42	42	D	0.341
	Likert scale score of 3	7	5	-	
	Likert scale score of 4	33	33		
	Likert scale score of 5	2	4		

A= ANOVA (Analysis of variance); B= Paired T-test C=Fisher's extract test; D=Regression analysis; POD= Post-operative day.
